# Mutation in Mg-Protoporphyrin IX Monomethyl Ester Cyclase Decreases Photosynthesis Capacity in Rice

**DOI:** 10.1371/journal.pone.0171118

**Published:** 2017-01-27

**Authors:** Xuexia Wang, Rongfeng Huang, Ruidang Quan

**Affiliations:** 1 Biotechnology Research Institute, Chinese Academy of Agricultural Sciences, Beijing, China; 2 National Key Facility of Crop Gene Resources and Genetic Improvement, Beijing, China; University of California - Davis, UNITED STATES

## Abstract

In photosynthesis, the pigments chlorophyll a/b absorb light energy to convert to chemical energy in chloroplasts. Though most enzymes of chlorophyll biosynthesis from glutamyl-tRNA to chlorophyll a/b have been identified, the exact composition and regulation of the multimeric enzyme Mg-protoporphyrin IX monomethyl ester cyclase (MPEC) is largely unknown. In this study, we isolated a rice pale-green leaf mutant *m167* with yellow-green leaf phenotype across the whole lifespan. Chlorophyll content decreases 43–51% and the granal stacks of chloroplasts becomes thinner in *m167*. Chlorophyll fluorescence parameters, including Fv/Fm (the maximum quantum efficiency of PSII) and quantum yield of PSII (Y(II)), were lower in *m167* than those in wild type plants (WT), and photosynthesis rate decreases 40% in leaves of *m167* mutant compared with WT plants, which lead to yield reduction in *m167*. Genetic analysis revealed that yellow-green leaf phenotype of *m167* is controlled by a single recessive genetic locus. By positional cloning, a single mutated locus, G286A (Alanine 96 to Threonine in protein), was found in the coding sequence of LOC_Os01g17170 (*Rice Copper Response Defect 1*, *OsCRD1*), encoding a putative subunit of MPEC. Expression profile analysis demonstrated that *OsCRD1* is mainly expressed in green tissues of rice. Sequence alignment analysis of CRD1 indicated that Alanine 96 is very conserved in all green plants and photosynthetic bacteria. OsCRD1 protein mainly locates in chloroplast and the point mutation A96T in OsCRD1 does not change its location. Therefore, Alanine96 of OsCRD1 might be fundamental for MPEC activity, mutation of which leads to deficiency in chlorophyll biosynthesis and chloroplast development and decreases photosynthetic capacity in rice.

## Introduction

Photosynthesis is the process of converting light energy to chemical energy and is the most important source of energy on the earth [1]. Chlorophyll (Chl) molecules harvest light energy for photosynthesis, so Chls are key cofactors for the photosynthetic apparatus [2].

The Chl biosynthesis pathway, including more than 17 enzymes in higher plants [3–7], comprises four distinct sections: common steps, heme/chlorophyll branch, chlorophyll cycle and chlorophyll breakdown [5]. The common steps start from 5-aminolevulinic acid (5-ALA) to protoporphyrin IX, which is a common precursor for Chl and heme biosynthesis [6, 7]. Chl branch starts from the insertion of Mg2+ into protoporphyrin IX by Mg chelatase to get Mg-protoporphyrin IX (MgP), followed by convertion to Mg-protoporphyrin IX monomethyl ester (MgPME) by a methyl transferase. Then, MgPME is used as a substrate for the Mg-protoporphyrin monomethyl ester cyclase (MPEC; EC 1.14.13.81) and creates protochlorophyllide (Pchlide) [8].

In the process of Chl biosynthesis, MPEC is one of the least understood enzymes. The first study on MPEC was carried out in cucumber (*Cucumis sativus*) [9]. MPEC is a multiprotein complex consisting of at least two subunits in cucumber [9]. Further research found that one of the soluble component of MPEC is over 30 kD [10]. An oxidative cyclase component of MPEC AcsF was firstly cloned from *Rubrivivax gelatinosus* [11]. From various organisms, using biochemical and genetic approaches, people have identified many *AcsF* homologs, such as *Chlamydomonas reinhardtii copper response defect 1* (*Crd1*) [12], *Pharbitis nil Leu zipper (PNZIP*) [13], *Epipremnum aureum ZIP* [14], *Nicotiana tabacum ZIP and Arabidopsis thaliana CHL27* [15], *Hordeum vulgare Xantha-l* [16]. In *Arabidopsis*, CHL27 involves in the conversion of MgPME to Pchlide, and leaves of either the antisense or T-DNA mutant are chlorotic or yellow, and chloroplast development is defected accompanied with PSI and PSII instability. [15, 17].

Low Chlorophyll Accumulation A (LCAA), with the Ycf54 domain, interacts with and stabilizes CHL27 protein in tobacco, which indicates LCAA may be another component of MPEC [18]. In *Synechocystis* YCF54-like protein (Ycf54), a potential component of MPEC identified by separate pulldown assay using two FLAG-tagged ACSF homologs as baits, is essential for the activity and stability of the oxidative cyclase [19]. Similarly, barley Ycf54, associating with XanL, also stimulates MPEC activity [20]. In addition, barley Viridis-k might be an additional membrane associated component of the MPEC [16, 20]. So far, the components of barley MPEC consist of a soluble protein and three membrane-bound components, Ycf54, Xanth-l and unknown Viridis-k [16, 20]. Therefore, MPEC is the only enzyme with unidentified components in chlorophyll biosynthesis.

In higher plants, all of mutants in MPEC subunits are chlorotic and dwarf [15–18, 20], indicating MPEC is essential for green plants. However, in most cereal crops like rice, MPEC has not been characterized genetically yet. In this study, we characterized a yellow-green leaf rice (*Oryza sativa*) mutant *m167* from a rice variety Kitaake. Young and mature leaves are yellow-green, chlorophyll content and photosynthesis rate decrease, and the chloroplast development is arrested in *m167* mutant. Map-based cloning revealed that there is a site-mutation in the coding region of *OsCRD1* gene encoding a putative subunit of MPEC.

## Materials and Methods

### Plant materials

The *m167* mutant was isolated from a EMS mutagenized population from the Japonica rice variety Kitaake. To construct the F_2_ mapping population, the yellow-green leaf rice mutant *m167* was crossed with rice varieties Zhefu802 and Dular, respectively. And the F_2_ population was planted in Langfang to collect yellow-green leaf segregating individuals for genotyping.

### Genetic analysis and map-based cloning

For genetic analysis in F_1_ and F_2_ populations, leaf color (yellow-green or green) of seedlings at 3-week was check with eye, and the F_2_ segregation ratios were analyzed by χ2 test.

DNA was extracted from leaves using the CTAB method. About 0.5 leaf tissues were ground in liquid nitrogen, added CTAB extraction buffer (2% CTAB, 0.1 M Tris-Cl pH8.0, 20 mM EDTA pH 8.0, 1.4 M NaCl, 1% PVP4000), and incubated at 65°C for 15 min. Then, each sample was added 1 volume of chloroform/isoamyl alcohol (96:4) and vortexed thoroughly. After centrifugation the aqueous layer were transferred to a new tube, and DNA was precipitated with 0.7 volume of isopropanol, washed with 0.5 mL 70% ethanol, and finally dissolved in 200 μL of water. Genotyping was performed by PCR using a set of SSR molecular markers from Gramene website (http://www.gramene.org/markers/)). Polymorphic SSR markers between the two parent lines were screened by the size of PCR products. Then the polymorphic SSR markers were used to identify the genotype of F2 progenies. Some of the SSR marker sequences were listed in S1 Table.

### Sequence analysis

Within the fine mapped chromosome region, candidate genes were screened according to the Rice Genome Annotation Project (http://rice.plantbiology.msu.edu/) and gene specific primers were designed accordingly (S1 Table). These primers were used to amplify the candidate genes from the *m167* mutant and wild type Kitaake. The PCR amplified products were sequenced to determine difference between *m167* and wild type plants.

Homologous sequences of *OsCRD1* were identified using the Blastp search program of the National Center for Biotechnology Information (http://www.ncbi.nlm.nih.gov) [21].

### RNA extraction and quantitative real-time PCR

Total RNA was extracted from various tissues of Kitaake and *m167* plants using Ultrapure RNA Kit (Cwbiotech, Beijing, China). Approximately 1 mg of total RNA from each sample was used for first-strand cDNA synthesis. For quantitative real-time RT-PCR, first strand cDNAs were used as templates in reactions using SYBR Green PCR Master Mix (Abm) according to the manufacturer’s instructions. OsActin gene was amplified as an internal control. Amplification of target genes were carried out using a real-time quantitative system (Bio-rad IQ5).

### Quantitative analysis of chlorophyll content, photosynthesis rate and chlorophyll fluorescence

For chlorophyll content determination, leaf tissues of 2-week-old and 2-month-old *m167* mutant and Kitaake (wild type) grown in the field were collected, and ground in ice-cold 80% acetone. Residual plant debris was removed by centrifugation at 8000 *g* for 10 min. Supernatants were analyzed with a visible spectrometer and chlorophyll contents were calculated as described previously [22].

To determine photosynthesis parameters, the plants of wild type and *m167* were planted in greenhouse for about 20 d before photosynthesis measurement. Photosyn- thesis (P) and transpiration (T) rates were measured using a portable photosynthesis system (LI-6400XT) in the morning (9 to 11 AM). All of the photosynthetic measurements were taken at a constant air flow rate of 500 μmol s-1. The concentration of CO2 was 400 μmol CO2 mol-1 using the system’s CO2 injector and the temperature was maintained at 30°C, and the photosynthetic photon flux density was 800 μmol (photon) m-2 s-1. Three measurements were made for each plant, and 5 plants were used for both the wild type and the mutant.

To determine chlorophyll fluorescence parameters, chlorophyll fluorescence in vivo was measured at room temperature on intact plant leaves at 4-week stage using a fluorometer (IMAGING-PAM, Waltz, Germany)[23]. Before measurements plants were dark adapted for at least 30 minutes at room temperature. Then a weak modulated measuring light was applied to register the minimal fluorescence yield (Fo). And then a saturating light pulse was applied to determine maximum fluorescence (Fm) and variable fluorescence (Fv). The photosystem II efficiency Y(II), quantum yield of light-induced non-photochemical quenching Y(NPQ), and quantum yield of non-regulated energy dissipation Y(NO) were calculated as following: Y(II) = (Fm’-F)/Fm’, NPQ = (Fm-Fm’)/Fm’, Y(NPQ) = 1-Y(II)-1/(NPQ+1+qL(Fm/Fo-1)), Y(NO) = 1/(NPQ+1+qL(Fm/Fo-1)). The chlorophyll fluorescence data was captured by a fluorometer IMAGING-PAM controlled by ImagingWin v2.41 software.

### Transmission electron microscopy (TEM) analysis

Leaf samples for TEM analysis were harvested from the wild type and *m167* at 20 d-stage. Fixation and polymerization of leaf samples were carried out as described previously [24]. The fresh leaf tissues were cut into pieces and fixed in a solution of 2% glutaraldehyde and further incubated overnight. After staining with uranyl acetate, tissues were further dehydrated in an ethanol series, and finally embedded in Spurr’s medium prior to ultrathin sectioning. Samples were stained again and examined with a transmission electron microscope (JEOL JEM-1230).

### Subcellular localization of OsCRD1 protein

The subcellular localization of OsCRD1 was was determined by transient expression of GFP fusion protein. The coding sequence of OsCRD1 was fused with GFP in-frame into a 35S-GFP vector. And then the GFP-OsCRD1 plasmid was introduced into rice green tissue protoplasts by PEG [25]. The transformed protoplast cells were examined by a confocal microscope.

### Determination of Chl precursors

The contents of chlorophyll precursors protoporphyrin IX (Proto IX), Mg-protoporphyrin IX / Mg-protoporphyrin IX monomethyl ester (MgP/MgPME) and protochlorophyllide (Pchlide) were determined according to the method described previously [26–30]. Briefly, the seedlings of WT and *m167* mutant were grown at 28°C in the dark for two weeks. Then, the intact leaves (approximately 4.0 g fresh weight) were incubated overnight at 22°C in darkness with 10 mM ALA in 50 mM phosphate buffer (pH 7.0). After incubation for 18 h, the leaves were extracted in 80% alkaline acetone under dim green light, then centrifuged for 10 min at 20 000 g, 4°C. The absorbance of the supernatants was determined with a spectrophotometer. The relative contents of chlorophyll precursors Proto IX, MgP/MgPME and Pchlide in *m167* mutant were calculated with those in WT were defined as 1.

### CRISPR/Cas9 mediated knock-out of OsCRD1 in wild type Kitaake plants

To confirm whether OsCRD1 is the candidate gene for *m167* mutant, we constructed the *OsCRD1* knock-out plants by CRISPR/Cas9 [31, 32]. Two gRNA targets were chosen, the first target was AGGAGGGAGAGCTCCATGG, and the second was GAA- GATGGTGATGTACCTC.

## Results

### Reduced chlorophyll accumulation, lower photosynthesis rate and delayed chloroplast development in *m167* mutant

**Fig 1:**
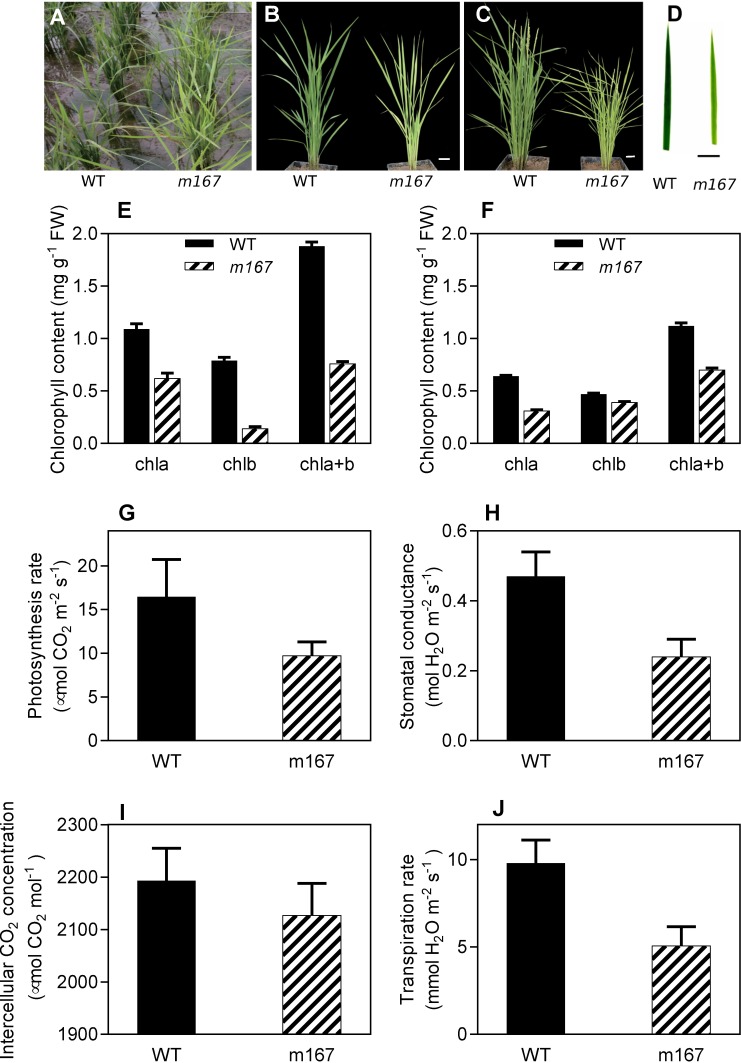
Physiological characterization of rice *m167* plants. A-D, *m167* shows yellow leaf at seedling (A and D), tilling (B), and heading stages(C). Bar = 5 cm. E-F, Leaf pigment contents of WT and *m167* at seedling (E) and heading stages (F). Chlorophyll contents data are mean ± SD (n = 3). G-J, Measurement of photosynthesis rate (G), stomatal conductance (H), intercellular CO2 concentration (I) and transpiration rate (J) of wild type (WT) and *m167* rice plants.

The *m167* mutant was isolated from a pool of EMS mutagenized *japonica* rice Kitaake. The *m167* mutant exhibits a yellow-green leaf phenotype throughout the whole developmental span (Fig 1A–1D). Plant height and yield of *m167* decreased by 26% and 49%, respectively, compared to that of wild type (Table 1).

**Table 1:** The agronomic traits of *m167* and wild type Kitaake plants.

	Plant height (cm)	Spike length (cm)	Grain number per spike	Tilling number	Seed- setting rate (%)	1000- grain weight (g)	Yield per plant (g)
Kitaake	71.7±4.0	13.3±1.2	59.6±9.2	19.8±3.2	94.6±3.0	20.1±0.1	22.5±4.2
*m167*	52.4±1.8	11.7±0.9	42.4±8.	16.6±2.3	77.8±4.8	19.4±0.2	11.4±4.3

Moreover, we examined chlorophyll content and photosynthesis rate of *m167* mutant. The content of Chl a and Chl b was significantly reduced in *m167* compared with the wild-type Kitaake. The Chl, Chl a and Chl b contents of *m167* were only about 57%, 18% and 40% of those of the wild type at seedling stage, respectively. And the Chl, Chl a and Chl b contents of *m167* were only about 49%, 82% and 63% of those of the wild type at heading stage, respectively. In addition, the Chl a/b ratio of *m167* increased at seedling stage but decreased at heading stage compared to that of wild type, indicating that the Chla/Chlb balance was destroyed in *m167* mutant (Fig 1E and 1F).

There was a decrease of photosynthesis, including net photosynthetic rate, stomatal conductance, intercellular carbon dioxide concentration and transpiration rate (Fig 1G–1J), suggesting that lower photosynthesis capacity in *m167* mutant. Lower photosynthesis capacity is consistent with lower grain yield. In addition, these results indicate that the regulation of stomatal aperture is affected by the mutation of CRD1 gene, implying that chlorophyll deficiency in chloroplasts of guard cells might malfunction the stomatal regulations.

**Fig 2:**
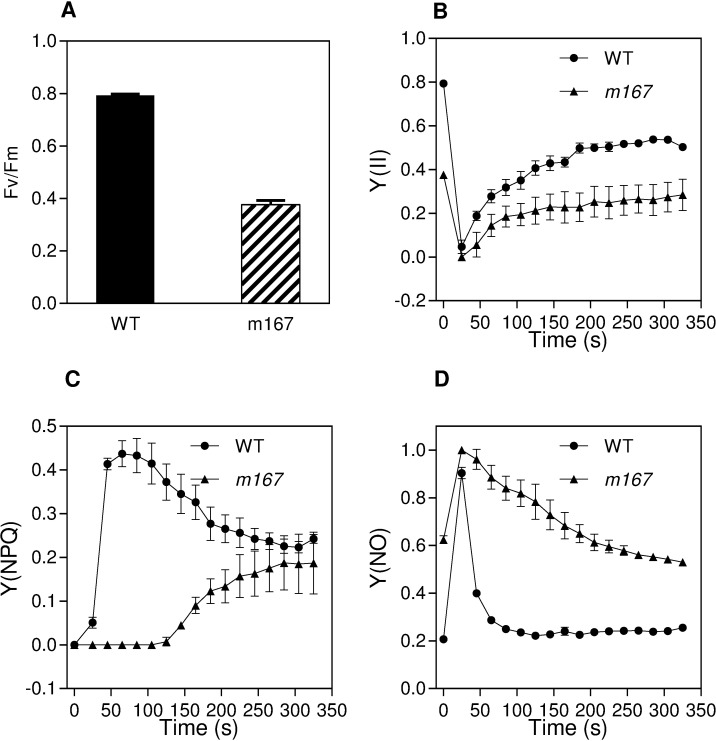
Chlorophyll fluorescence parameters of *m167* and WT plants. (A) Fv/Fm of leaves of *m167* and wild type plants. (B-D) Chlorophyll fluorescence kinetics including quantum yield of PSII Y(II) (B), quantum yield of light-induced non-photochemical quenching Y(NPQ) (C), and quantum yield of non-regulated energy dissipation Y(NO) (D) in leaves of *m167* and wild type plants. Data are means ± SD (n = 10).

To investigate the photosynthetic capacity of photosystem II (PSII) in the *m167* mutant, we determined the chlorophyll fluorescence data of rice seedlings at 4-week stage by a chlorophyll fluorometer (IMAGING-PAM, Heinz Walz GmbH). Fv/Fm (the maximum quantum efficiency of PSII) in *m167* was about half of that in WT (Fig 2A), indicating the maximum photosynthetic capacity of PSII was damaged in *m167* by lacking of chlorophyll. In addition, we captured chlorophyll fluorescence parameters during illumination with actinic light at 186 μmol m-2 s-1 in a time-dependent manner (Fig 2B–2D). Quantum yield of PSII (Y(II)) was significantly lower in *m167* than that in WT (Fig 2B), and Y(II) increased more gently over time in *m167* than that in WT. In addition, quantum yield of light-induced non-photochemical quenching Y(NPQ) was also lower in *m167* than that in WT (Fig 2C), but quantum yield of non-regulated energy dissipation Y(NO) was much higher in *m167* than that in WT (Fig 2D). Y(II) and Y(NPQ) are decreased and Y(NO) is increased in *m167*, indicating the absorbed light energy are dissipated by Y(NO) in *m167* mutant. Therefore, the photosynthetic capacity of PSII was greatly damaged in *m167* mutant.

**Fig 3:**
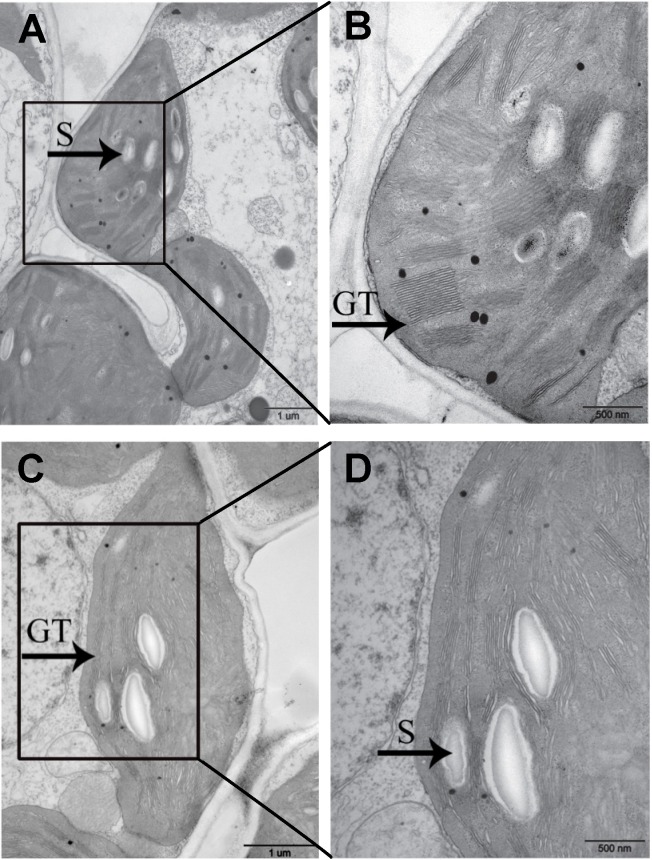
Transmission electron micrographs of chloroplasts. Transmission electron micrographs of chloroplasts in WT (A and B) and *m167* (C and D) plants. S and GT indicate starch granules and granal thylakoids, respectively.

To investigate if the chloroplast development was affected in *m167* mutant, we investigated the ultrastructure of plastids in *m167* mutant and WT plants at 3-week-old seedlings using transmission electron microscopy (Fig 3). Granal stacks in the *m167* mutant appeared less dense and lacked granal membranes compared to those of WT in developing leaves. Therefore, in *m167* mutant the chloroplast development is partially impaired.

### Map-based cloning of *m167* mutation site

To map the mutation site in *m167* mutant, two F_1_ and F_2_ populations were constructed by crossing *m167* to Dular and Zhefu802, respectively. Then, we calculated the ratio of green:yellow-green plants in F_1_ and F_2_ generations (Table 2). The segregation ratio indicates that the mutation of a single recessive genetic locus might cause the yellow-green leaf phenotype in the *m167* mutant.

**Table 2:** Segregation of F_1_ and F_2_ populations from two crosses.

	F_1_	F_2_			
	Percentage of green plants	No. of yellow- green plants	No. of green plants	Ratio	χ2
*m167*×Dular	100%	194	634	1:3.27	0.316
*m167*×Zhefu802	100%	105	370	1:3.52	2.196

**Fig 4:**
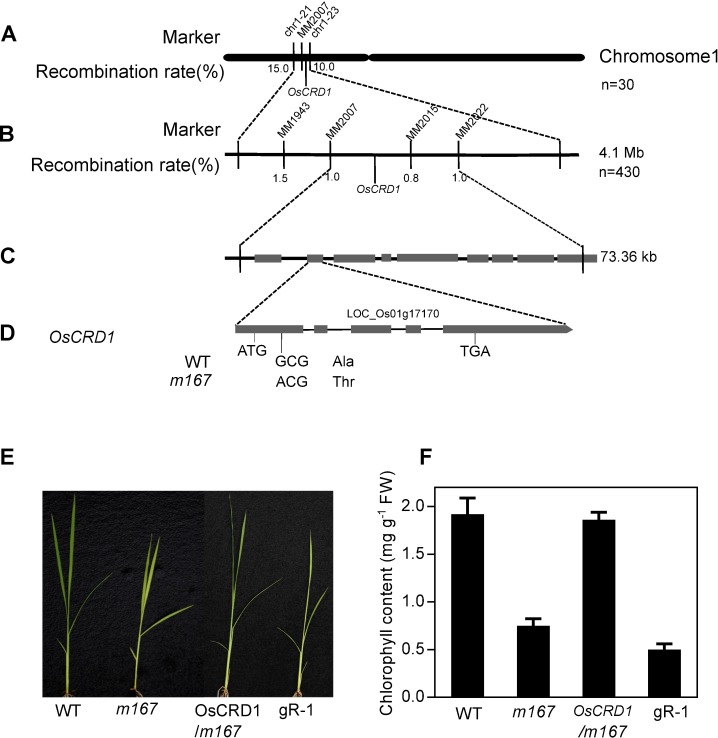
Map-based cloning of OsCRD1 in *m167*. A, The candidate gene was mapped to a region between SSR markers Chr1-21 and Chr1-23 on chromosome 1. B, The target region was narrowed to a 73.36-kb region between SSR markers MM2007 and MM2022. C, There are nine ORFs between MM2007 and MM2022, including LOC_Os01g17160, LOC_Os01g17170, LOC_Os01g17180, LOC_Os01g17190, LOC_Os01g17214, LOC_Os01g17240, LOC_Os01g17250, LOC_Os01g17260, LOC_Os01g17279, respectively(from left to right). D, Structure of OsCRD1 gene. ATG and TGA represent the start and stop codons, respectively. Black boxes indicate the exons. A single G to A substitution in the first exon in *m167*. E. Seedlings of *m167* complemented with wild type OsCRD1 genomic sequence (OsCRD/*m167*) and edited in OsCRD1 coing sequence of wild type by CRISPR-Cas9 method (2gR-1). F. Chlorophyll concentration of seedlings shown in E. Pictures are representative photos of 3 independent transgenic lines with similar results. Values are means *±* SD with 3 independent transgenic lines.

The genetic mapping of *m167* mutation was performed using the F_2_ population from *m167*×Dular cross. In initial mapping of the *m167* target gene, we used approximately 200 SSR markers evenly distributing on 12 chromosomes. The mutation was initially mapped between the markers Chr 1–21 and Chr 1–23 on the short arm of chromosome 1 (Chr 1 represents chromosome 1 and the number 21 and 23 represent the serial number of markers). And then we enlarged the population for fine mapping using 430 segregated recessive individuals from F_2_ population of *m167*×Dular. The mutation was located between markers MM2007 and MM2022 (http://archive.gramene.org/markers/microsat/). The mutation site was subsequently narrowed to a 73.36 kb region. Within this chromosomal region, nine open reading frames (ORFs) have been predicted according to the Rice Genome Annotation Project (http://rice.plantbiology.msu.edu) (S2 Table). All genes within this region were amplified and sequenced in *m167* and wild type Kitaake plants. A single nucleotide G-to-A substitution was found at position 286 in the coding region in the first exon of LOC_Os01g17170 (*OsCRD1*) in *m167* (Fig 4). This substitution changes amino acid 96 from alanine (A) to threonine (T) in OsCRD1 protein. No sequence variations were detected in the genomic sequences of the other 8 candidates.

To verify the mutation of *OsCRD1* conferring yellow-green leaf in *m167*, we transferred OsCRD1 genomic sequence to *m167* mutants, and found the pale green phenotype of *m167* was rescued. In addition, we also found that knocking out OsCRD1 in wild type rice by CRISPR-Cas9 method caused leaf color to pale green (Fig 4E and 4F, S1 Fig).

All the above results demonstrate that the mutation in *OsCRD1* leads to leaf color change in *m167* mutant.

### OsCRD1 belongs to a subunit of magnesium-protoporphyrin IX monomethyl ester cyclase

Blast search in the genome database revealed that *OsCRD1* is a single-copy gene in rice. In green plants, the number of CRD1 homologous genes alters in different species, with a single copy in most of crops but up to 17 copies in *Ostreococcus tauri* (GreenPhl v4, http://www.greenphyl.org/cgi-bin/family.cgi?p=id&family_id=2552#tab-famcomp). *OsCRD1* has an open reading frame (ORF) of 1227 bp encoding a 408-amino acid protein with molecular mass 47.3 KDa.

Multiple amino acid sequence alignment indicated that OsCRD1 has higher sequence similarity in all species. The MPEC subunit encoded by *OsCRD1* has more than 80% identity with other orthologs in green algae, bryophyte and higher green plants (S2 Fig), including CHL27 in *Arabidopsis*, suggesting that it is a highly conserved protein and might be essential for photosynthesis. Sequence alignment indicated that the mutation of *OsCRD1* in *m167* is highly conserved in different green plants and rice varieties (S2 and S3 Figs).

### *OsCRD1* is mainly expressed in green tissues

**Fig 5:**
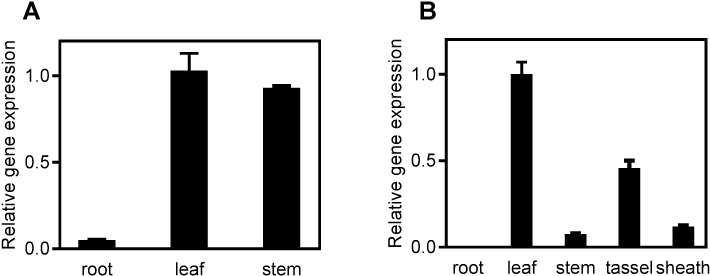
Expression analysis of *OsCRD1*. A. *OsCRD1* gene expression at seedling stage. B. *OsCRD1* gene expression at heading stage. Expression of *OsCRD1* in leaves was analyzed using quantitative RT-PCR. Osactin gene was used as an internal control. Data are means ± SD (n = 3).

Quantitative real-time PCR analysis showed that *OsCRD1* is mainly expressed in green tissues including stem, leaf, tassel and sheath but not in root (Fig 5). The expression in leaf was relatively high, while the expression in root was almost undetectable or at a very low level. A single nucleotide G-to-A substitution in *OsCRD1* leads to its expression downregulation in *m167* compared with WT plants (S4 Fig).

### OsCRD1 protein localizes in chloroplast

**Fig 6:**
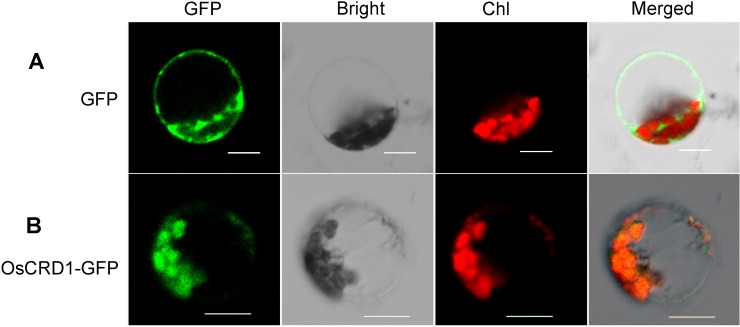
Subcellular localization of OsCRD1 in rice protoplast. A, Fluorescence localization of free GFP in rice protoplast. B, Fluorescence localization of OsCRD1-GFP fused protein in rice protoplast.

To determine the subcellular location of OsCRD1 protein, we constructed *OsCRD1- GFP* vector for transient expression in rice protoplasts mediated by PEG. Confocal microscopy analysis of OsCRD1-GFP location showed that OsCRD1 is localized in chloroplast (Fig 6). To test whether OsCRD1 Ala 96 to Thr (OsCRD1A96T) mutation in *m167* affected the subcellular localization, we also performed a transient expression analysis of the OsCRD1A96T-GFP fusion protein. OsCRD1A96T mutant proteins were localized in chloroplast (S5 Fig), indicating the OsCRD1A96T mutation does not affect protein localization.

### Mutation in OsCRD1 impaired MPEC activity

**Fig 7:**
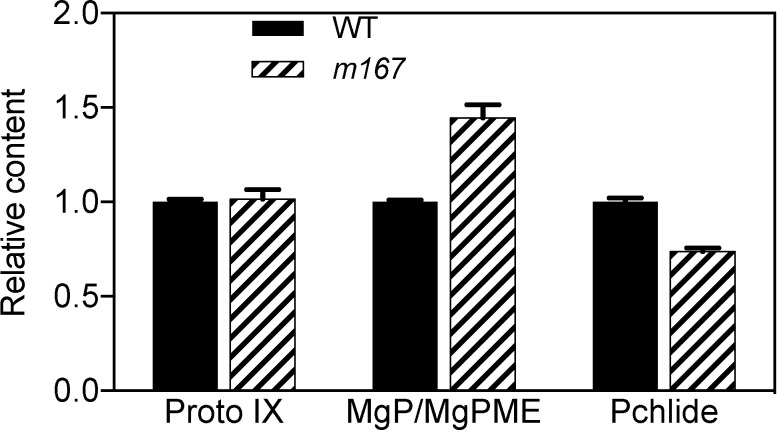
Analysis of chlorophyll (Chl) intermediates Proto IX, MgP/MgPME and Pchlide in WT and *m167* mutant.

The yellow-green leaf phenotype of *m167* suggested that Chl biosynthesis in *m167* mutant might be damaged. So we determined the intermediates Proto IX, MgP/MgPME and Pchlide in Chl biosynthesis (Fig 7). Compared to WT, Proto IX was slightly increased in *m167* mutant, and MgP/MgPME were increased by 44% in *m167* mutant. However, Pchlide was decreased by about 30% in *m167* mutant (Fig 7). Therefore, the increase of MgP/MgPME and decrease of Pchlide in *m167* mutant indicate that the cyclase activity of MPEC was damaged in *m167* mutant possibly by a mutation in OsCRD1.

## Discussion

Chlorophylls are essential for photosynthesis and plant growth. Chlorophyll biosynthesis is a highly coordinated process that is executed via a series of cooperative reactions catalyzed by numerous enzymes. However, only several genes have been studied in rice. In this study, we characterized a yellow-green leaf rice mutant *m167*. By map-based cloning, we cloned the mutation gene *OsCRD1*, encoding a putative subunit of MPEC, the most enigmatic enzyme in chlorophyll biosynthesis.

Similar to previous identified abnormal leaf color mutants [33–38], chloroplast development in *m167* is impaired, which indicates that the chlorophyll synthesis is co-regulated with chloroplast development [39]. Chlorophyll molecules absorb light in photosystems embedded in the chloroplast thylakoid membranes, therefore, lack of chlorophyll might impair the photosynthetic performance. Whether there is any specific regulatory function of CRD1 or MPEC enzyme on photosynthetic functions is an interesting topic to be addressed in the future.

In *Arabidopsis*, leaves of *gun4* mutant range from albino to pale green to yellow-green under normal growth conditions [40–42]. In *Arabidopsis* GUN4 promotes the activity of Mg-chelatase, upstream of MPEC in chlorophyll biosynthesis pathway [42, 43]. Whether OsGUN4 regulates MPEC in rice is to be investigated in future studies.

Previous studies revealed that MPEC is a multimeric enzyme in green algae and plants, and its activity requires several components. In our study, we found that yellow-green plants of *m167* did not accumulate Pchlide when fed 5-ALA in the dark as WT plants, indicating OsCRD1 might participate in Chl biosynthesis. In *m167* mutant, MgPME accumulated and Pchlide decreased, which implied that *m167* is deficient in the cyclase [11, 15]. We incubated the purified recombinant OsCRD1 from *E*. *coli* with the substrate MProtoME in the reaction buffer, but did not detect the production of Pchlide after incubation, possibly resulting from the lack of other components of MPEC [9–11]. Thus, the biochemical property of MPEC needs to be confirmed, and its regulation mechanism is still unknown in rice yet.

## Supporting Information

S1 Fig Sequencing confirmation for complementation of *m167* with WT OsCRD1 genomic sequence (A) and knocking out of *OsCRD1* in wild type Kitaake (B).
(PDF)

S2 Fig Sequence alignment of OsCRD1 protein and its related proteins. (PDF)

S3 Fig Sequence analysis of *OsCRD1* in different rice varieties. (PDF)

S4 Fig Expression of *OsCRD1* gene in 2-week seedlings of WT and *m167*. (PDF)

S5 Fig Subcellular localization of OsCRD1A96T in rice protoplasts. (PDF)

S1 Table Sequences of the primers used in this study. (DOC)

S2 Table Nine candidate genes annotated in mapping region. (DOC)
