# Supplementary material for: Mutation in Mg-Protoporphyrin IX Monomethyl Ester Cyclase Decreases Photosynthesis Capacity in Rice
Source: PLoS One. 2017 Jan 27;12(1):e0171118. doi: 10.1371/journal.pone.0171118 (PMC5271374; doi:10.1371/journal.pone.0171118)
Supplement: S1 Fig — Sequencing confirmation for complementation of m167 with WT OsCRD1 genomic sequence (A) and knocking out of OsCRD1 in wild type Kitaake (B). (PDF) [file pone.0171118.s001.pdf]

A

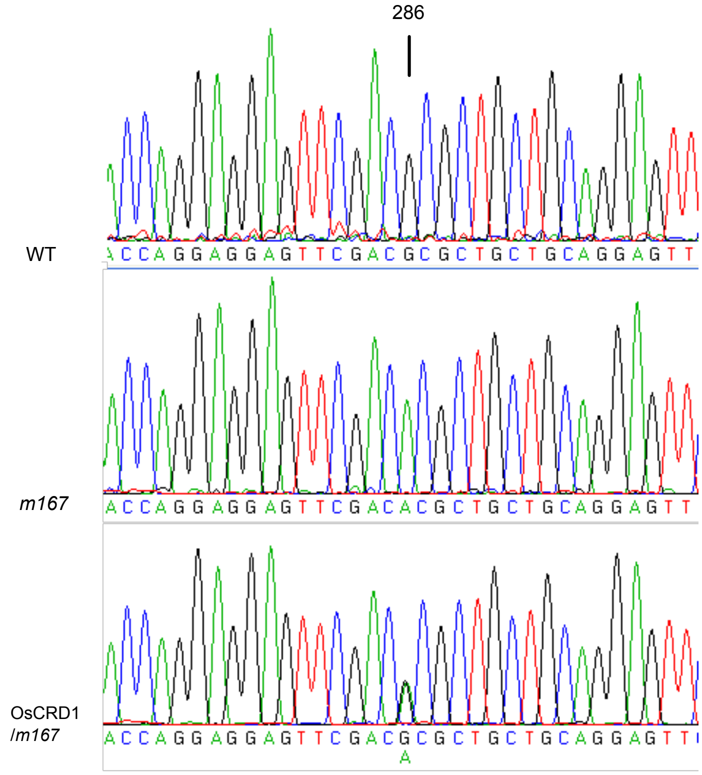

B

	target 1	target 2
wt	GGCCTCCTCCGCCATGGAGCTCTCCCTCCT	TACTGGAGGTACATCACCATCTTCAGGCAC
OsCRD1-2gR-1	GGCCTCCTCCGCC - -GGAGCTCTCCCTCCT	TACTGGAGGTACATCAC - - - - TTCAGGCAC

S1 Fig. Sequencing confirmation for complementation of *m167* with WT *OsCRD1* genomic sequence (A) and knocking out of *OsCRD1* in WT rice (B).
