# Supplementary material for: Mutation in Mg-Protoporphyrin IX Monomethyl Ester Cyclase Decreases Photosynthesis Capacity in Rice
Source: PLoS One. 2017 Jan 27;12(1):e0171118. doi: 10.1371/journal.pone.0171118 (PMC5271374; doi:10.1371/journal.pone.0171118)
Supplement: S2 Fig — (PDF) [file pone.0171118.s002.pdf]

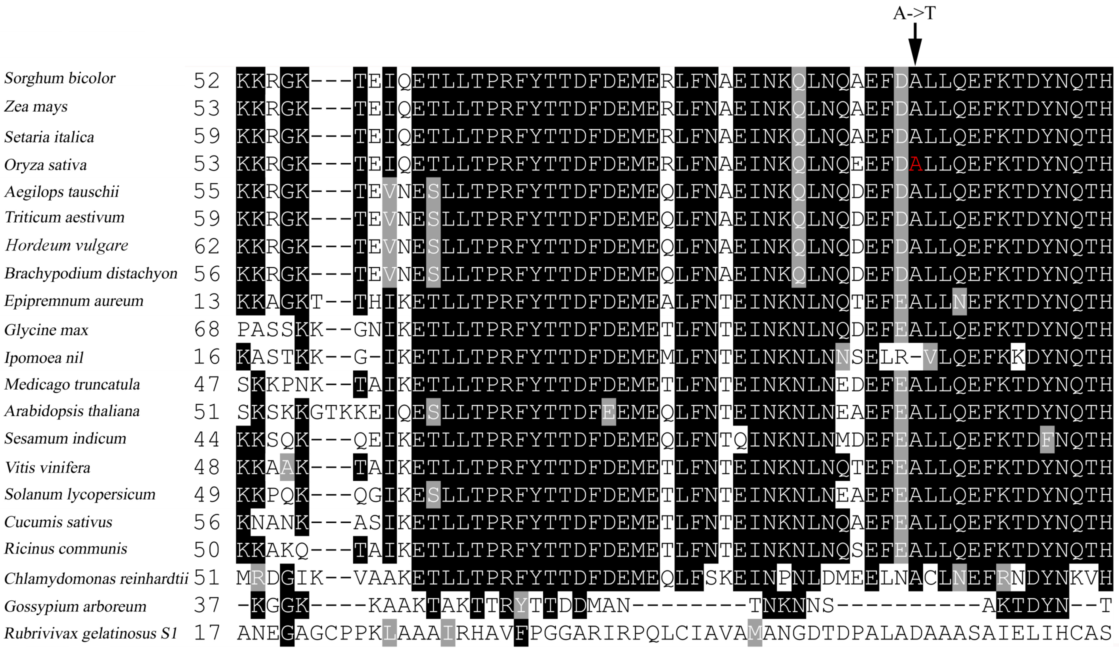

S2 Fig. Sequence alignment of OsCRD1 protein and its related proteins.

Alignment of the OsCRD1 protein and its related proteins from other plants by NCBI-BLAST. Conserved residues or similarity of the aligned sequences are depicted in the consensus sequence with lower case. Accession numbers: XP_002457655.1 for *Sorghum bicolor*, XP 008655474.1 for *Zea mays*, XP_004967679.1 for *Setaria italic*, NP_001042745.1 for *Oryza sativa*, EMT08710 for *Aegilops tauschii*, CDM82987 for *Triticum aestivum*, Q5EFU4.1 for *Xanthal*, XP_003567276 for *Brachypodium distachyon*, ACX54448 for *Epipremnum aureum*, XP_003521093for *Glycine max*, AAB19120.1 for *Ipomoea nil*, XP_003624856.1 for *Medicago truncatula*, NP_191253.1 for *Arabidopsis thaliana*, XP_011070036 for *Sesamum indicum*, XP_002283892.1 for *Vitis vinifera*, XP_004249099.1 for *Solanum lycopersicum*, XP_004144646 for *Cucumis sativus*, XP_002523677 for *Ricinus communis*, XP_001692557.1 for *Chlamydomonas reinhardtii*,

KHG30795.1 for *Gossypium arboretum*, AAO93113.1 for *Rubrivivax gelatinosus* S1.
