# Supplementary material for: Mutation in Mg-Protoporphyrin IX Monomethyl Ester Cyclase Decreases Photosynthesis Capacity in Rice
Source: PLoS One. 2017 Jan 27;12(1):e0171118. doi: 10.1371/journal.pone.0171118 (PMC5271374; doi:10.1371/journal.pone.0171118)
Supplement: S3 Fig — (PDF) [file pone.0171118.s003.pdf]

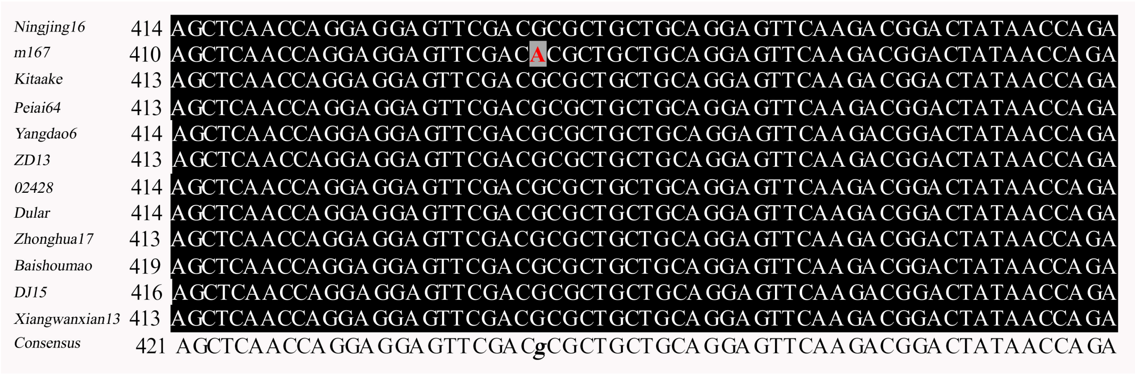

S3 Fig. Sequence analysis of OsCRD1 in different rice varieties.
