# Supplementary material for: Mutation in Mg-Protoporphyrin IX Monomethyl Ester Cyclase Decreases Photosynthesis Capacity in Rice
Source: PLoS One. 2017 Jan 27;12(1):e0171118. doi: 10.1371/journal.pone.0171118 (PMC5271374; doi:10.1371/journal.pone.0171118)
Supplement: S4 Fig — (PDF) [file pone.0171118.s004.pdf]

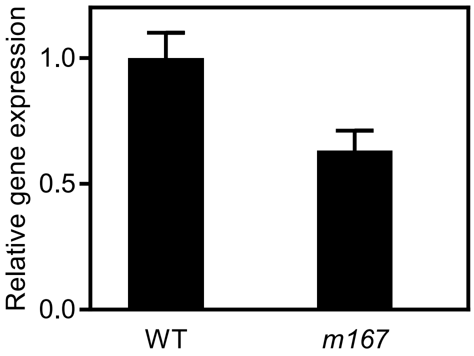

S4 Fig. Expression of *OsCRD1* gene in 2-week seedlings of WT and *m167*.

Expression of *OsCRD1* in leaves was analyzed using quantitative RT-PCR. *Actin* gene was used as a control. Data are means ± SD ($n = 3$).
