# Supplementary figures and images for: Mutation in Mg-Protoporphyrin IX Monomethyl Ester Cyclase Decreases Photosynthesis Capacity in Rice
Source: PLoS One. 2017 Jan 27;12(1):e0171118. doi: 10.1371/journal.pone.0171118 (PMC5271374; doi:10.1371/journal.pone.0171118)

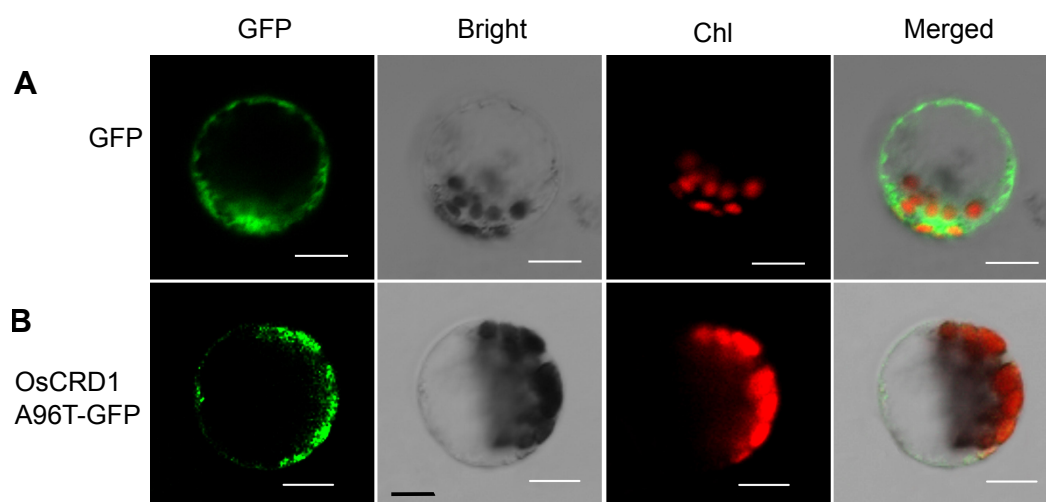

1

2

**S5 Fig. Subcellular localization of OsCRD1A96T in rice protoplasts.**

Supplement: S5 Fig — (PDF) [file pone.0171118.s005.pdf]
