# Supplementary material for: Mutation in Mg-Protoporphyrin IX Monomethyl Ester Cyclase Decreases Photosynthesis Capacity in Rice
Source: PLoS One. 2017 Jan 27;12(1):e0171118. doi: 10.1371/journal.pone.0171118 (PMC5271374; doi:10.1371/journal.pone.0171118)
Supplement: S1 Table — (DOC) [file pone.0171118.s006.doc]

| **S1 Table. Sequences of the primers used in the article** | | | | |
| --- | --- | --- | --- | --- |
| Mapping primers | | | | |
| Marker | Forward sequence (5’-3’) | | | Reverse sequence (5’-3’) |
| chr1-21 | TACTATATTTGTTTTTCATGGGAT | | | AGCACAAAGAATACTACTACCTTTA |
| chr1-23 | AAAGCAGGTTTTCCTCCTCC | | | CCCATGTGCAATGTGTCTTC |
| MM1943 | GAAATGGACTCGCTCCTAAACTGG | | | ACGAACTAGAGCATGGGCACTCC |
| MM2007 | CGCGGTTAATGTCATCTGATTGG | | | CCATACTTCGAGATCCAAGACTGACC |
| MM2022 | AGTACGATTTCTGTCAGCGTTGC | | | TGAAAGGAGTAGCCAGAGAAAGC |
| MM2015 | ATGCAAACTCCCTCCATCC | | | GAAGTTTACCTGCCTGTGGC |
| Quantitative PCR primers | | | | |
| Primer | | Forward sequence (5’-3’) | | Reverse sequence (5’-3’) |
| OsCRD1 | | GCGGATGGTGGAGGAGA | | TGCCGATGGTGTTGACG |
| OsActin | | GATGACCCAGATCATGTTTG | | GGGCGATGTAGGAAAGC |
| Vector construction primers | | | | |
| Primer | | | Sequence (5’-3’) | |
| GFP-F | | | GGACTAGTATGGCCTCCTCCGCCATGGA | |
| GFP-R | | | GCTCTAGAGTAGACAAGCTGGGGCTCGA | |
