# Supplementary material for: Mutation in Mg-Protoporphyrin IX Monomethyl Ester Cyclase Decreases Photosynthesis Capacity in Rice
Source: PLoS One. 2017 Jan 27;12(1):e0171118. doi: 10.1371/journal.pone.0171118 (PMC5271374; doi:10.1371/journal.pone.0171118)
Supplement: S2 Table — (DOC) [file pone.0171118.s007.doc]

**S2 Table. Nine candidate genes annotated in mapping region**

| Gene ID | Physical location | Gene function annotation |
| --- | --- | --- |
| LOC_Os01g17160 | Chr 1: 9866424-9870864 | Subtilisin homologue |
| LOC_Os01g17170 | Chr 1: 9874326-9876951 | magnesium-protoporphyrin IX monomethyl ester cyclase,chloroplast precursor |
| LOC_Os01g17180 | Chr 1: 9878631-9885134 | proteasome subunit |
| LOC_Os01g17190 | Chr 1: 9887626-9889328 | OsCam3 - Calmodulin |
| LOC_Os01g17214 | Chr 1: 9889548-9902738 | major facilitator superfamily antiporter |
| LOC_Os01g17240 | Chr 1: 9913390-9917318 | transporter, major facilitator family |
| LOC_Os01g17250 | Chr 1: 9917474-9921595 | Brassinosteroid insensitive 1-associated receptor kinase 1 precursor |
| LOC_Os01g17260 | Chr 1: 9928866-9935981 | transcription factor |
| LOC_Os01g17279 | Chr 1:9936753-9947940 | exonuclease |
